# Administration of Immunoglobulins in SARS-CoV-2-Positive Patient Is Associated With Fast Clinical and Radiological Healing: Case Report

**DOI:** 10.3389/fmed.2020.00388

**Published:** 2020-07-16

**Authors:** Novella Carannante, Giuseppe Fiorentino, Antonio Corcione, Raffaele Di Sarno, Micaela Spatarella, Nicola Maturo, Fiorentino Fragranza, Pierpaolo Di Micco

**Affiliations:** ^1^Emergency Infectious Disease p.o. Cotugno-Monaldi, A.O. dei Colli, Naples, Italy; ^2^UOC Medicina, Ospedale Fatebenefratelli di Naples, Naples, Italy

**Keywords:** PENTAGLOBIN, immunoglobulin, SARS-CoV2, COVID 19, pneumonia, case report

## Abstract

Polyclonal preparation of IgM as an adjuvant therapy has been reported as a relevant immunomodulant therapy in several infectious diseases, exhibiting, in most cases, improvement of the clinical course. No drug has demonstrated therapeutic efficacy for COVID-19. Immunomodulatory treatment with hydroxychloroquine and biologics as tocilizumab, in fact, has not proven to show satisfactory results in several reports. We therefore treated a selected patient with interstitial multifocal pneumonia, positive to COVID-19, with polyclonal preparation of immunoglobulins as an adjuvant therapy, obtaining in few days clinical remission and improvements in radiological findings. Based on this case report, we suggest that clinical trials are conducted to test the efficacy and safety of polyclonal immunoglobulins for adjunctive therapy of COVID-19.

## Background

After its recent identification in China, COVID-19 may appear with different clinical features (1); it may be, in fact, asymptomatic, and it may induce isolated pneumonia as well as multifocal bilateral interstitial pneumonia, which may lead to severe acute respiratory syndrome (SARS) (2). Admission to ICU for affected patients is usually possible for those with elevated associated mortality, as recently testified by the Italian COVID-19 outbreak (3).

Compared to other coronavirus infections such as SARS and MERS, the clinical course of COVID-19 may be longer than 20 days because associate impairments of the cytokine network are able to induce immunopathological damages in lung or other localizations (4).

So, the international medical community tried different approaches to counteract this complex disease. In this way, antiviral drugs, immunomodulatory drugs, biologics, and steroids have been tested in different subjects in order to improve their outcomes and in particular to induce regression of lung infiltrates and to obtain immunological power vs. the virus (3).

We here report our clinical experience in one selected case of COVID-19 with polyclonal preparation of IgM as adjuvant therapy (i.e., PENTAGLOBIN) in addition to antiviral and immunomodulant therapy and to antithrombotic prophylaxis; the case has been interesting because the patient showed a fast clinical and radiological improvement in 10 days.

## Case History

**Figure 1:**
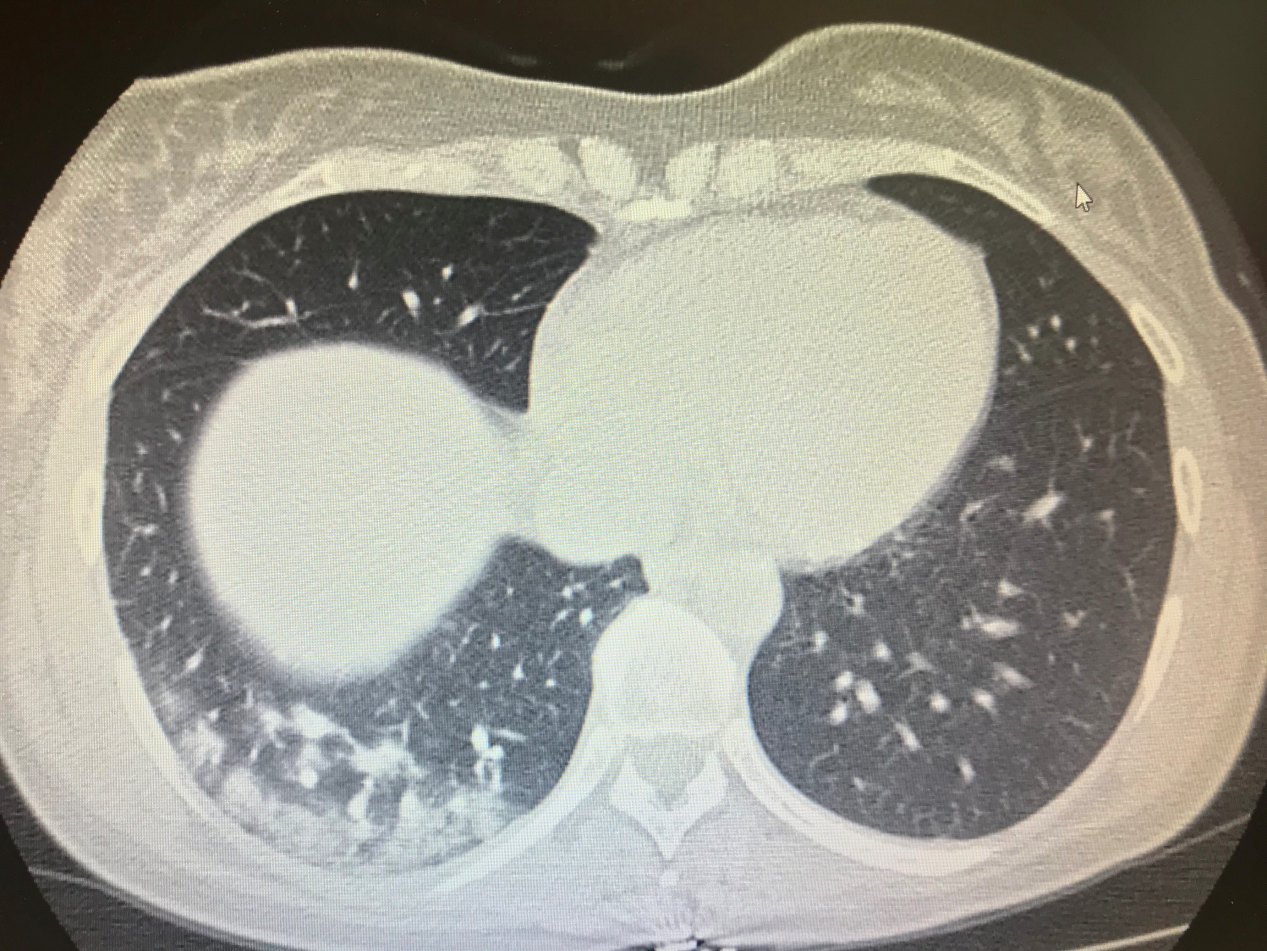
Lung CT scan with evidence of multifocal pneaumonia.

On April 4, 2020, a female patient with cough and fever was referred to our emergency department for infectious diseases because these symptoms were considered as suspected for COVID-19; the symptoms appeared the day before her admission to emergency department. She was 43 years old and a carrier of inherited thrombophilia without previous thrombotic episodes, and she was not taking any type of antithrombotic drug. She was immediately addressed to a COVID-19 emergency ward because she had been in contact with a COVID-19 positive patient 7 days before. A physical examination and routine blood samples for infectious diseases were immediately performed, and due to the anamnesis of cough, fever, and suspected COVID-19, a chest CT scan was consequently performed, revealing interstitial bilateral pneumonia with several ground glass areas (Figure 1). A treatment with several drugs based on hydroxychloroquine 200 mg twice daily, azithromycin 500 mg daily, enoxaparin 4,000 UI twice daily, and Darunavir/Cobicistat 800 mg daily was planned as a specific antiviral treatment; Vitamin C 1.5 gr daily and Ceftaroline 600 twice daily were added the following day to prevent bacterial superinfection and to add antioxidant action. Oxygen support with a ventimask with a fraction of inspired oxygen (FiO2) 0.60% was associated.

Temperature, pulsoximetry, blood pressure, heart rate, and blood samples were routinely checked; in particular, hemocrome with leukocyte formula, C reactive protein, fibrinogen, d-dimer, LDH, and interleukin-6 were frequently checked, and their trend is reported in Table 1. In order to look for levels of IgM and IgG anti COVID-19, Sierological tests were performed with an ELISA method and chemiluminescence immunoassays (Elecsys, Anti-SARS-CoV2, Roche, Italy; LIAISON SARS-CoV-2, DiaSorin S.p.A., Italy). Results are reported in Table 1. A nasopharyngeal swab (Real-time PCR, DiaSorin Molecular Simplexa™ COVID-19 Direct assay, DiaSorin S.p.A., Italy) was performed to look for the qualitative detection of nucleic acid from SARS-CoV-2 and confirmed that the patient was affected by COVID-19.

**Table 1:** Laboratory tests for the studied patient.

**Test**	**Date 04/04/2020**	**Date 08/04/2020**	**11/04/2020**	**18/04/2020**	**02/05/2020**
WBC (MMCUBE)	4.900 (MMCUBE)	3390 (MMCUBE)	4100 (MMCUBE)	8720 (MMCUBE)	7950 (MMCUBE)
Lymphocites %	17	26	40	50	45
CRP (MCG\DL)	1.5	5	3.5	<0.5	<0.5
IL-6 (UI\ml)	13	8.8	5.6	2.6	
P/F	300	200	200	>350	Not tested
IgM anti-COVID19 (UI\ml)	0.54	Not tested	0.41	Not tested	0.48
IgG anti-COVID19 (UI\ml)	0.29	Not tested	76.30	Not tested	78.50
SWAB (real time PCR)	Positive	Not tested	Negative	Negative	Negative

On the day April 8, 2020, due to worsening of lung performance testified by clinical features and with arterial-blood gas analysis (P/F 200 with respiratory rate of 24 acts p.m.), we began therapy with intravenous polyclonal immunoglobulins (i.e., PENTAGLOBIN, Biotest, Germany). We chose this kind of drug because the personal anamnesis of inherited thrombophilia that could be associated to increased rate of venous thromboembolism during prolonged hospitalization and/or during COVID-19 infection. PENTAGLOBIN is a pharmacological drug that consists of different classes of standard immunoglobulins (i.e., 6 mg of IgM, 6 mg of IgA, and 38 mg of IgG). This arrangement is considered a highly rich IgM preparation. PENTAGLOBIN was administered at the dose of 5 ml/kg/daily for 3 days by an intravenous way for a time of 12 h (continuous intravenous infusion at 28 ml\h).

This drug showed good tolerance for the patient, and a good therapeutic response was associated and testified obtaining progressive reduction of the inflammatory markers CRP, IL6, and fibrinogen (Table 1); common side effects of PENTAGLOBIN, such as hemolytic anemia and kidney failure, were frequently monitored and not detected.

**Figure 2:**
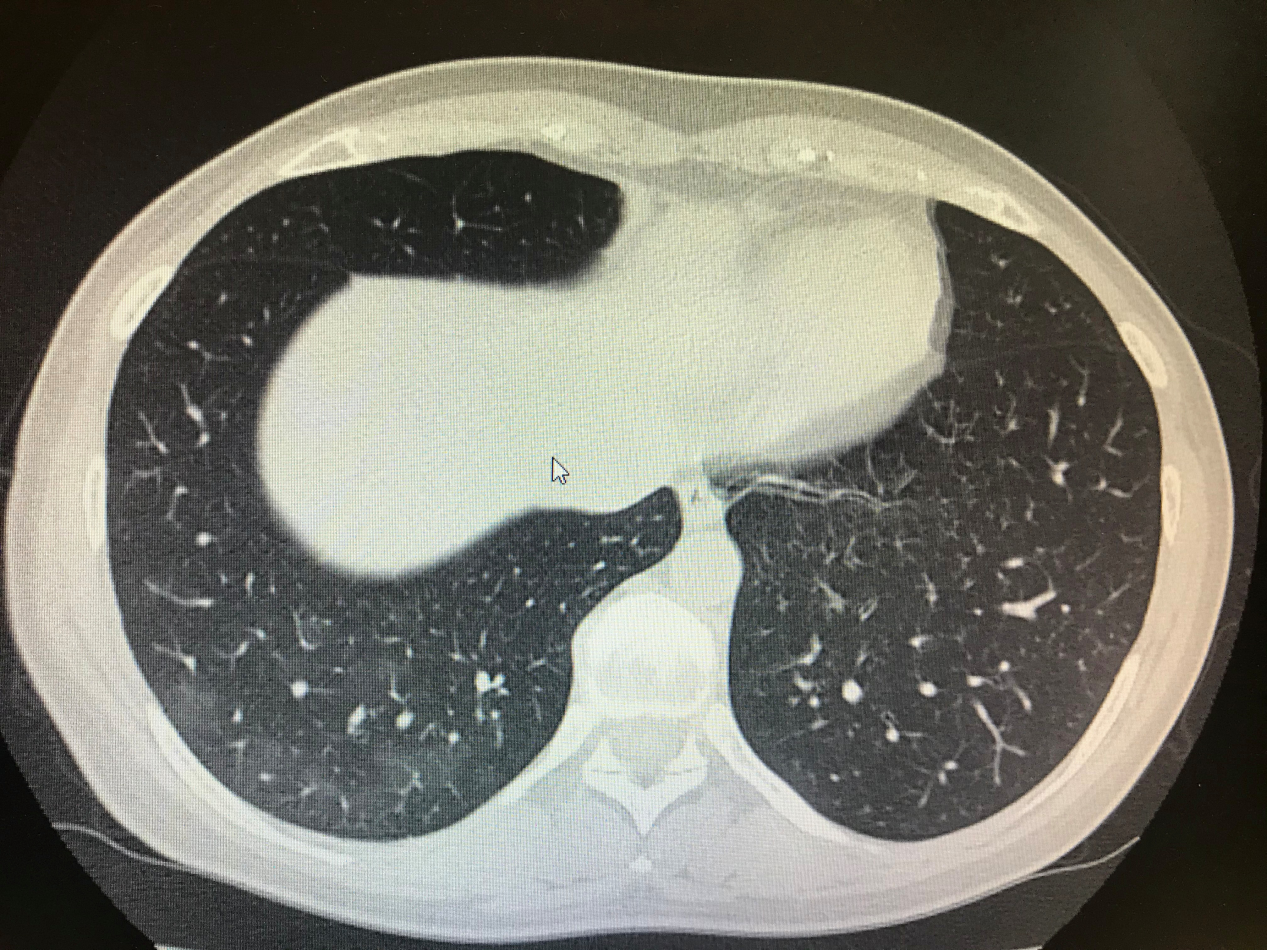
Lung CT scan after treatment with pneumonia resolution.

As previously reported, on the admission day, we also tested IgG anti COVID-19, which showed increased levels at baseline (i.e., 0.54 UI/ml; normal values 0.00), and IgM anti COVID-19 were also positive at baseline (i.e., 0.29 UI|\mL normal values 0.00) (Elecsys, Anti-SARS-CoV2, Roche, Italy; LIAISON SARS-CoV-2, DiaSorin S.p.A., Italy). Of course, these values were recorded before to start PENTAGLOBIN. After the administration of PENTAGLOBIN, we checked levels of IgG and IgM anti COVID-19 in the patient again (i.e., 7 days later), and we found that her levels were increased, and they were also associated with a serum conversion of immunoglobulins: IgG anti COVID-19 increased to 73.30 UI/ml IgM, and anti COVID-19 increased to 0.41 UI\ml (Table 1). Furthermore, associated with these immunological improvements, we also recorded clinical amelioration with reduction of fever and improvement of lung performance. Arterial-blood gas analysis confirmed this trend with the following data: P/F > 350 without oxygen support (respiratory rate of 19 acts p.m.). Progressive improvements were also found with a lung chest CT scan on April 18, 2020: a marked reduction in lung thickening was found (Figure 2).

Furthermore, 7 days after the beginning of therapy with PENTAGLOBIN, and then, 10 days after the clinical onset, the patient also had viral clearance: two consecutive nasopharyngeal swab results were negative (Real-time PCR, DiaSorin Molecular Simplexa™ COVID-19 Direct assay, DiaSorin S.p.A., Italy), and so she was dismissed in spontaneous breath without oxygen support and with fine systemic condition; enoxaparin 4,000 U daily was suggested for a further 14 days as home care treatment. A clinical and laboratory follow-up was planned 15 days after hospital discharge, and a further nasopharyngeal swab (Real-time PCR, DiaSorin Molecular Simplexa™ COVID-19 Direct assay, DiaSorin S.p.A., Italy) tested negative; levels of IgG and IgM anti COVID-19 were tested again, and the previous trend was confirmed by results, as IgG anti COVID-19 was increased to 78.50 UI/ml and IgM anti COVID-19 increased to 0.45 UI\ml (Table 1).

Verbal informed consent was given by the patient to describe her clinical experience; in this way, we have been awarded with a recognition for “Best practice” from our Health management and from the ethics committee of AO dei Colli.

## Discussion

The COVID-19 pandemic represents the greatest global public health of recent times. There are no univocal treatments suggested by guidelines for this disease. No treatment, in fact, has been demonstrated as effective on a significant population, and this therapeutic difficulty is also related to the length of disease and to the multiple pathophysiological mechanisms that are induced by viral actions and by the host-immune response. Recently, several authors reported that the immunopathological phase occurs after 10–15 days of the onset of the disease (4). Clinical benefits with antiviral, steroid, and immunomodulatory drugs (e.g., remdesivir, glucocorticoids, and hydroxychloroquine, respectively) (5) have not been conclusive in clinical trials because they seem to not be effective on the cytokine storms that present during the disease. Furthermore, the use of biological drugs, such as tocilizumab, that play a relevant role in the cytokine network has not reported univocal results (5–7).

Based on the capacity of polyclonal preparation of immunoglobulins (i.e., PENTAGLOBIN) as an adjuvant therapy in other infectious diseases, we therefore tried to select patients that may benefit from this drug. The adjuvant action of polyclonal immunoglobulins is due to its support of physiological immune defense, as previously reported, in meningococcal disease and in septic shock (8, 9). We thus tried to administer this kind of drug in a selected patient in order to improve her interstitial pneumonia and to speed up the host immunoglobulins production (8, 9). Our attempt was also supported by other case reports in the literature that rarely underlined that the use of intravenous immunoglobulins as preventive care of respiratory distress syndrome was associated to satisfactory clinical and radiological improvements. In this way, someone also suggested the intravenous use of immunoglobulins in selected cases of COVID-19 in which the immune response should be reinforced (10).

Actually, the use of intravenous immunoglobulins is suggested by guidelines for the substitutive therapy of secondary inherited or acquired immunodeficiencies (11), while there are not current available guidelines for their routinely use during other infectious diseases.

Based on our clinical experiences in other infectious diseases reported in other selected cases in the literature, therefore, we tried to administer PENTAGLOBIN in the reported patient in order to induce a right and improved regulation of immune system and to speed up her healing. Clinical, laboratory and radiological CT findings, in fact, suggested that the administration of this kind of immunoglobulin may improve the clinical course of COVID-19.

Furthermore, in our reported experience, the utility of this treatment has been stronger than expected because the drug was administered starting on days 7–8 from clinical onset, which is commonly more frequently associated with immunologically impaired functions in COVID-19.

In conclusion, our experience is compatible with the concept that the early use of intravenous immunoglobulins such as PENTAGLOBIN for 3 consecutive days can slow down the cytokines' hyperactivation and can induce an important immunological support in the healing of COVID-19 pneumonia (12).

Of course, several criticisms may be raised for our case. First of all, this clinical and therapeutic approach should be tested in randomized controlled trials as we are trying to do: the clinical goal should be to always administer the drug within 7–12 days, monitoring clinical, laboratory, and radiological findings in order to suggest a potential therapeutic protocol for other patients; on the other hand, being a treatment that may be associated with several side effects and one that is not cheap *per se*, a thorough evaluation of each patient should be performed, as in our case, in order to evaluate the risk\benefit ratio.

## Data Availability Statement

All datasets generated for this study are included in the article/supplementary material.

## Ethics Statement

Written informed consent was obtained from the individual for the publication of any potentially identifiable images or data included in this article.

## Author Contributions

All authors listed have made a substantial, direct and intellectual contribution to the work, and approved it for publication.

## Conflict of Interest

The authors declare that the research was conducted in the absence of any commercial or financial relationships that could be construed as a potential conflict of interest.
